# Simplified hypertension screening approaches with low misclassification and high efficiency in the United States, Nepal, and India

**DOI:** 10.1111/jch.14299

**Published:** 2021-09-03

**Authors:** Olive Tang, Minghao Kou, Yifei Lu, Edgar R. Miller, Tammy Brady, Cheryl Dennison‐Himmelfarb, Arun More, Dinesh Neupane, Lawrence Appel, Kunihiro Matsushita

**Affiliations:** ^1^ Johns Hopkins University Baltimore MD USA; ^2^ University of North Carolina Chapel Hill NC USA; ^3^ Rural Health Progress Trust Osmanabad India; ^4^ Nepal Development Society Bharatpur Nepal

**Keywords:** blood pressure, blood pressure measurement/monitoring, classification, high blood pressure, hypertension

## Abstract

Standard triplicate blood pressure (BP) measurements pose time barriers to hypertension screening, especially in resource‐limited settings. We assessed the implications of simplified approaches using fewer measurements with adults (≥18 years old) not using anti‐hypertensive medications from the US National Health and Nutrition Examination Survey 1999‐2016 (*n* = 30 614), and two datasets from May Measurement Month 2017‐2018 (*n* = 14 795 for Nepal and *n* = 6 771 for India). We evaluated the proportion of misclassification of hypertension when employing the following simplified approaches: using only 1st BP, only 2nd BP, 2nd if 1st BP in a given range (otherwise using 1st), and average of 1st and 2nd BP. Hypertension was defined as average of 2nd and 3rd systolic BP ≥140 and/or diastolic BP ≥90 mm Hg. Using only the 1st BP, the proportion of missed hypertension ranged from 8.2%–12.1% and overidentified hypertension from 4.3%–9.1%. Using only 2nd BP reduced the misclassification considerably (corresponding estimates, 4.9%–6.4% for missed hypertension and 2.0%–4.4% for overidentified hypertension) but needed 2nd BP in all participants. Using 2nd BP if 1st BP ≥130/80 demonstrated similar estimates of missed hypertension (3.8%–8.1%) and overidentified hypertension (2.0%–3.9%), but only required a 2nd BP in 33.8%–59.8% of participants. In conclusion, a simplified approach utilizing 1st BP supplemented by 2nd BP in some individuals has low misclassification rates and requires approximately half of the total number of measurements compared to the standard approach, and thus can facilitate screening in resource‐constrained settings.

## 1 INTRODUCTION

The global morbidity and mortality due to hypertension continue to rise,^1^ with a greater burden, and lower awareness and control, in low‐ and middle‐income countries compared to high‐income countries.^2^, ^3^ To address this hypertension management gap, several global organizations, including the International Society of Hypertension (ISH) and the World Hypertension League (WHL), are encouraging and implementing hypertension screening programs worldwide.^4^, ^5^ These programs screen a remarkably large number of individuals (eg, ISH/WHL screened ~4 million individuals in the first three years),^6^, ^7^, ^8^ but the standard approach of triplicate blood pressure (BP) measurement^9^ poses time constraints.

A few previous studies explored whether the 1st BP reading is sufficient to identify hypertension, and these studies generally concluded that repeated BP measurements remained important.^10^, ^11^, ^12^ However, using data from a US community‐based cohort, we recently reported that mainly relying on the 1st BP measurement supplemented by 2nd measurement only when the 1st BP is higher a prespecified value may be a reasonable approach to facilitate higher volume screening with minimal misclassification using the same resources.^13^ Nonetheless, this concept has not been systematically explored in multi‐country datasets. Thus, we assessed the degree of misclassification when using simplified approaches (ie, using only 1st and/or 2nd BP) compared to the standard BP measurement approach with triplicate measurements in datasets from three countries: United States, Nepal, and India. We characterized four parameters for each approach: missed hypertension (no hypertension based on a simplified approach, but hypertension according to the standard approach), overidentified hypertension (hypertension based on a simplified approach, but no hypertension according to the standard approach), the proportion of individuals requiring a 2nd BP measurement, and the total number of required BP measurements.

## 2 MATERIAL AND METHODS

### 2.1 Study population

Data from the United States were obtained from the National Health and Nutrition Examination Survey (NHANES) 1999‐2016.^14^ NHANES recruited participants from across the United States using multi‐stage random sampling approaches in 2‐year cycles.^15^

Data from Nepal and India were obtained from May Measurement Month (MMM) 2017 and 2018.^16^, ^17^ MMM participants consisted of volunteer individuals interested in undergoing BP screening. Further details of MMM are described elsewhere.^16^, ^17^

We included all adults aged 18 years or older who did not self‐report using hypertension medications and had triplicate BP data (Figure S1). For NHANES‐USA and MMM‐Nepal, those missing data on medication use were excluded. Given missing data on medication use in most participants in MMM‐India, we considered missing information on medication use as not taking hypertension medication and retained them in our study population. We also excluded participants with any of the following conditions: age >120 years, systolic BP (SBP) <30 mmHg or >200 mmHg, diastolic BP (DBP) <30 mmHg or >150 mmHg, and the recorded DBP greater than the recorded SBP. Our final analytic population included 30 614 participants for NHANES‐USA, 14 795 participants for MMM‐Nepal, and 6 771 participants for MMM‐India.

### 2.2 Blood pressure measurement protocol

NHANES trained and certified all physicians who did BP measurements for the study. BP was measured following procedures developed by the American Heart Association with a Baumanometer calibrated mercury true gravity wall model sphygmomanometer, with a 2‐mm increment markings interval. Appropriate arm cuff sizes (child, standard, large, and plastic thigh cuff) were determined by measured arm circumference (corresponding arm circumference, 17–21.9 cm, 22–29.9 cm, 30–37.9 cm, and 38–47.9 cm).^18^ After determining the peak inflation level, the 1st measurement was obtained after at least 5‐min rest, with two subsequent measurements obtained at least 30 s apart.^18^ Hypertension medication use was self‐reported by participants.

In MMM, the standard protocol recommended use of automated devices donated by Omron (eg, HEM‐7120‐AP); however, if an Omron device was unavailable, a manual sphygmomanometer was used. In total, 87.3% of readings were taken using Omron devices globally.^7^ Appropriate arm cuff sizes (regular, large, extra‐large, and pediatric cuff) were determined by measured arm circumferences (corresponding arm circumference, <32 cm, 32–42 cm, >42 cm, and <20 cm).^19^ Triplicate BP readings were conducted by trained volunteers and obtained with 1‐min rest between measurements after at least 5‐min rest. More details regarding the MMM protocol have been published previously.^7^, ^16^

### 2.3 Standard and simplified BP approaches

Based on the recommendation by the World Health Organization (WHO),^9^ we considered the average of 2nd and 3rd BP as the standard reference, and hypertension was defined as SBP ≥140 and/or DBP ≥90 mmHg.^20^ We explored several simplified approaches requiring fewer than three BP measurements, such as only 1st BP, only 2nd BP, or the average of 1st and 2nd BP. We also evaluated approaches mainly relying on 1st BP measurement but using 2nd measurement when 1st measurement was higher than a certain threshold. We investigated all combinations of SBP of 130, 135, and 140 mmHg and DBP of 80, 85, and 90 mmHg as potential thresholds. After that, we added upper thresholds (145, 150, and 155 mmHg for SBP and 85, 90, and 95 mmHg for DBP) to the prior approach and used 2nd BP measurement when 1st measurement was between lower and upper thresholds.

### 2.4 Statistical analysis

**FIGURE 1:**
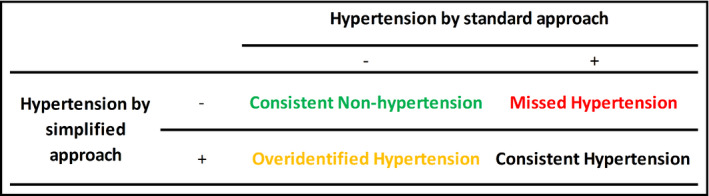
Blood pressure cross‐categories in combinations of simplified vs. standard approaches

For each dataset, we summarized age, sex, mean standard BP, and hypertensive status. As estimation of the prevalence of hypertension in the population was not our objective, we did not apply sample weighting when analyzing NHANES data. We visually assessed the prevalence of hypertension based on the standard approach by categories of 1st SBP and DBP, separately. Multinomial logistic regression models were then used to assess potential correlates (age, sex, and 1st BP measurement) of missed and overidentified hypertension. The sensitivity analysis was also conducted among three age groups: 18–40, 41–59, and ≥60 years old. Then, as the main analysis, we quantified missed hypertension (no hypertension with simplified approach among hypertension by the standard approach) and overidentified hypertension (hypertension with simplified approach among no hypertension by the standard approach) (Figure 1) as well as the proportion of individuals requiring 2nd BP measurement and total number of measurements by each simplified approach.^13^ All analyses were performed with Stata version 15.0, and a *p*‐value <0.05 was considered statistically significant.

## 3 RESULTS

Participants in NHANES‐USA and MMM‐Nepal were on average younger than those in MMM‐India. In all three study populations, the mean of the 1st SBP measurement was highest, and the mean of the 3rd SBP was lowest. The same pattern was seen for DBP in NHANES‐USA and MMM‐Nepal, whereas 2nd and 3rd DBP was lowest and highest, respectively, in MMM‐India. Within each dataset, the prevalence of hypertension was lowest when using the standard approach (ie, the average of 2nd and 3rd BP) and highest when relying on 1st BP (Table 1).

**TABLE 1:** Characteristics of study populations

	NHANES‐USA	MMM‐Nepal	MMM‐India
N	30 614	14 795	6 771
Age, %			
18–29	29.9	42.8	21.3
30–39	20.3	22.7	18.4
40–49	18.0	16.0	18.0
50–59	12.9	10.0	15.8
60–69	10.2	5.0	15.8
70+	8.7	3.5	10.7
Male, %	49.7	52.8	41.6
Systolic blood pressure, mean(SD), mmHg			
First	120.2 (16.6)	123.1 (17.2)	129.6 (18.8)
Second	119.3 (16.2)	120.9 (16.6)	128.4 (18.3)
Third	118.7 (15.9)	119.3 (16.1)	128.0 (17.7)
Average of first + second	119.8 (16.1)	122.0 (16.4)	129.0 (18.0)
Average of second + third^b^	119.0 (15.8)	120.1 (16.0)	128.2 (17.4)
Diastolic blood pressure, mean(SD), mmHg			
First	70.1 (11.4)	78.6 (11.6)	80.1 (11.7)
Second	69.7 (11.3)	77.6 (11.4)	79.9 (11.3)
Third	69.6 (11.4)	76.5 (11.4)	80.3 (11.1)
Average of first + second	69.9 (11.1)	78.1 (11.1)	80.0 (11.0)
Average of second + third^b^	69.7 (11.1)	77.1 (11.1)	80.1 (10.7)
Hypertension^a^ prevalence, no. (%)			
First	14.1	24.1	30.6
Second	12.7	20.2	28.5
Third	12.1	17.9	28.5
Average of first + second	12.4	20.7	27.8
Average of second + third^b^	11.5	17.8	27.0

Abbreviation: BP, blood pressure; MMM, May Measurement Month; NHANES, National Health and Nutrition Examination Survey.

^a^ BP ≥140/90 mmHg.

^b^ standard.

Only 0.2%–2.0% of participants with 1st SBP <120 mmHg were subsequently hypertensive according to the standard approach in all three datasets (Figure S2). On the other end of the spectrum, among those participants with 1st SBP ≥150 mmHg, only 3.9%–9.4% were subsequently non‐hypertensive according to the standard approach. When using only 1st BP, the highest proportion of participants with missed hypertension have BP level right below the threshold of hypertension (140/90 mmHg). Similarly, overidentified hypertension most likely happened to individuals with BP right above the threshold (Figure S3). Multinominal logistic regression confirmed these findings and identified 1st BP reading as the most potent correlate of missed and overidentified hypertension (Table 2). Specifically, 1st BP right below the threshold of hypertension had the highest odds of missed hypertension in all three datasets, whereas 1st BP right above this threshold had the highest odds of overidentified hypertension. Older age and male gender were associated with increased odds of missed and overidentified hypertension in some datasets, but the magnitude of association was much smaller than 1st BP reading. Consistent findings were observed among three age groups (Table S1).

**TABLE 2:** Adjusted odds ratios (95% confidence intervals) of missed hypertension and overidentified hypertension when using 1st blood pressure only based on multinomial logistic regression

		NHANES‐USA		MMM‐Nepal		MMM‐India
Missed hypertension	^a^cases/total		^a^cases/total		^a^cases/total	
Age, per 5 years		1.07 (1.04, 1.11)*		1.09 (1.04, 1.14)*		1.04 (0.99, 1.08)
Male		1.24 (0.98, 1.57)		1.45 (1.07, 1.96)*		1.25 (0.95, 1.65)
First SBP, <120 mmHg	15/16 432	ref	28/6 334	ref	34/2 033	ref
120 ≤ SBP <130 mmHg	76/6 556	8.08 (4.60, 14.21)*	60/3 257	2.28 (1.42, 3.68)*	74/1 571	1.87 (1.19, 2.96)*
130 ≤ SBP <140 mmHg	247/3 316	45.53 (26.49, 78.26)*	127/2 076	7.76 (4.94, 12.18)*	114/1 066	4.35 (2.77, 6.83)*
140 ≤ SBP <150 mmHg	0/1 061	–	0/768		0/530	
SBP ≥150 mmHg	0/1 758	–	0/990		0/883	
First DBP, <70 mmHg	56/13 784	ref	9/2 863	ref	24/1 128	ref
70 ≤ DBP <80 mmHg	92/9836	1.40 (1.00, 1.97)*	36/4 720	1.53 (0.73, 3.23)	46/2 107	0.76 (0.45, 1.28)
80 ≤ DBP <90 mmHg	190/4 331	4.53 (3.31, 6.20)*	170/3 933	5.59 (2.76, 11.35)*	152/1 793	2.37 (1.44, 3.91)*
90 ≤ DBP <100 mmHg	0/892	–	0/1268		0/696	
DBP ≥100 mmHg	0/280	–	0/641		0/359	
Overidentified hypertension						
Age, per 5 years		1.08 (1.05, 1.10)*		0.88 (0.85, 0.90)*		0.96 (0.93, 1.00)
Male		0.97 (0.83, 1.12)		0.96 (0.83, 1.12)		0.89 (0.72, 1.10)
First SBP, <120 mmHg	43/16 432	ref	63/6 334	ref	17/2 033	ref
120 ≤ SBP <130 mmHg	134/6 556	5.89 (4.14, 8.39)*	186/3 257	3.76 (2.76, 5.13)*	34/1 571	2.68 (1.45, 4.96)*
130 ≤ SBP <140 mmHg	135/3 316	8.15 (5.64, 11.77)*	227/2 076	5.01 (3.63, 6.89)*	99/1 066	9.73 (5.51, 17.19)*
140 ≤ SBP <150 mmHg	769/1 061	160.66 (114.52, 225.39)*	576/768	40.26 (29.43, 55.07)*	249/530	47.20 (26.78, 83.16)*
SBP ≥150 mmHg	72/1 758	4.84 (3.14, 7.46)*	103/990	5.09 (3.51, 7.39)*	67/883	8.82 (4.75, 16.38)*
First DBP, <70 mmHg	244/13 784	ref	39/2 863	ref	25/1 128	ref
70 ≤ DBP <80 mmHg	300/9836	1.03 (0.85, 1.25)	130/4 720	1.30 (0.89, 1.90)	77/2 107	0.84 (0.51, 1.38)
80 ≤ DBP <90 mmHg	246/4 331	1.10 (0.89, 1.36)	306/3 933	2.06 (1.42, 2.99)*	135/1 793	0.92 (0.56, 1.50)
90 ≤ DBP <100 mmHg	362/892	9.31 (7.34, 11.80)*	624/1268	10.71 (7.36, 15.59)*	214/696	2.21 (1.35, 3.61)*
DBP ≥100 mmHg	1/280	0.03 (0.00, 0.22)	56/641	1.39 (0.87, 2.22)	15/359	0.37 (0.18, 0.75)*

Note

Model adjusted for age, sex, SBP and DBP. SBP = systolic blood pressure, DBP = diastolic blood pressure. Hypertension was defined as SBP ≥140 mmHg and/or DBP ≥90 mmHg.

^a^ cases = number of individuals who were missed hypertension or overidentified hypertension when using 1st blood pressure only, total = number of individuals who were correctly diagnosed as hypertensive of normotensive when using 1st blood pressure only.

* Indicates statistical significance.

**FIGURE 2:**
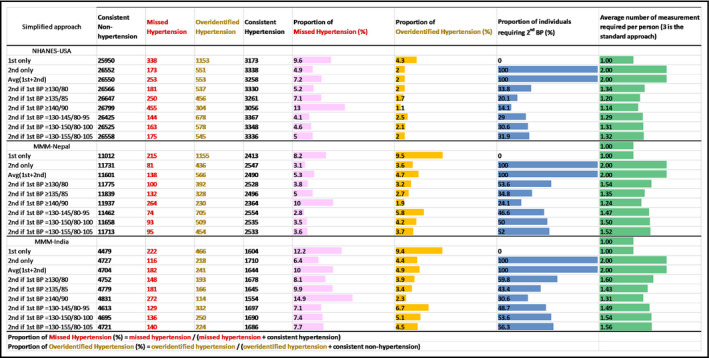
Misclassification and efficiency by the simplified approaches vs. standard approach. Color bars indicate degree of misclassification, the proportion of individuals requiring 2nd BP measurement (out of 100%), or average number of measurements required per person (3 is the standard approach). BP indicates blood pressure; MMM, May Measurement Month; NHANES, National Health and Nutrition Examination Survey

When relying on 1st BP, 9.6%, 8.2%, and 12.2% of hypertensive cases according to the standard approach were missed in NHANES‐USA, MMM‐Nepal, and MMM‐India, respectively (the pink bars in Figure 2). The proportion of overidentified hypertension with 1st BP was 4.3%, 9.5%, and 9.4%, respectively (the orange bars in Figure 2). A lower proportion of misclassification was observed when using only the 2nd BP measurement (4.9%, 3.1%, and 6.4% missed hypertension; 2.0%, 3.6%, and 4.4% overidentified hypertension). The average of the 1st and 2nd BP measurement was not better than using only 2nd BP regarding both missed and overidentified hypertension. By definition, these two approaches required measuring 2nd BP in all participants (100% in the blue bars of Figure 2).

Restricted use of 2nd BP measurement according to 1st BP value yielded similar misclassification and smaller proportion of population requiring a 2nd BP compared to employing a single 2nd BP for all (Figure 2). For example, using 2nd BP when the 1st BP was ≥130/80 mmHg, the proportion of missed hypertension was 5.2%, 3.8%, and 8.1% in NHANES‐USA, MMM‐Nepal, and MMM‐India, respectively, whereas the proportion of overidentified hypertension was 2.0%, 3.2%, and 3.9%, respectively. Under this scenario, there would be 33.8%, 53.6%, and 59.8% of individuals requiring a 2nd measurement in each of the three datasets. These correspond to approximately half of a total number of BP measurements (green bars of Figure 2). Using a higher threshold (135/85 or 140/90) led to fewer individuals requiring a 2nd BP reading and less overidentified cases but more missed hypertension cases. Additionally imposing an upper threshold to 130/80 mmHg further reduced the proportion of participants requiring 2nd measurement but did not reduce the misclassification of hypertension. Age‐stratified results (≤ vs. >50 years) largely demonstrated the same pattern (Figure S4).

## 4 DISCUSSION

Based on data from NHANES in the USA and the MMM initiative in Nepal and India, we observed that simplified BP screening approaches leveraging the 1st measurement and supplementing with a 2nd measurement in a subsample could be used to improve the efficiency of BP screening with minimal misclassification. More complex approaches, such as adding an upper threshold, could improve screening efficiency by decreasing the percentage of individuals needing a 2nd measurement but this approach did not confer an improvement in hypertensive status classification. Therefore, if the goal is to reduce the burden on medical resources while keeping low misclassification rates, using 2nd BP if 1st BP ≥130/80 mmHg is a good choice. However, the optimal threshold and approach may differ based on the resource setting and the local assessments regarding the trade‐offs between sensitivity and specificity of the screening.

Surveillance guidelines from the WHO recommend using the average of the 2nd and 3rd BP measurements to screen for hypertension.^9^ Multiple measurements are intended to accommodate the natural physiologic variability of BP,^21^ while discarding the 1st measurement appears intended to compensate for the tendency of the initial BP measurement to be higher than subsequent measurements. This phenomenon has been attributed to several potential causes, including response to physician measurement^22^ or reactive hyperemia.^23^ Consistent with these concepts, we observed that the 1st SBP in all three datasets was on average higher than the 2nd or 3rd measurements.

Our results suggest that classifying hypertension based solely on the 1st BP measurement was not reliable, with nearly 10% missed and overidentified cases, even though this approach is most efficient in terms of the number of BP measurement. Using the 2nd measurement only or the average of 1st and 2nd BP measurement instead, yielded less misclassification compared to when relying solely on the 1st measurement. However, this approach requires taking two BP measurements for everyone, which may still be burdensome in resource‐limited settings. Also, discarding the 1st measurement may have a deleterious impact on measurement quality. For example, if individuals measuring BP know that the 1st BP will not be used, they may measure the 1st BP with less care, which may influence the quality of 2nd BP measurement as well.

Our study suggests that incorporating the 2nd measurement in a subsample based on the 1st BP reading may be a reasonable alternative approach for hypertension screening. For example, in the NHANES‐USA dataset, using the 2nd BP only when the 1st BP was ≥130/80 mmHg reduced the total number of measurements by more than 50% (average of 1.34 measurements per individual) and resulted in minimal misclassification, with 2.0% overidentified hypertension and 5.2% missed hypertension when compared to the standard of employing triplicate measurements for all. With this approach, the misclassification rates were slightly higher and the efficiency was lower (namely, a higher proportion required a 2nd BP measurement) in the MMM‐India and MMM‐Nepal datasets, but the general patterns were similar across the three datasets.

A higher threshold of 1st BP (eg, 135/85 mmHg) as a trigger for 2nd BP measurement led to an even smaller proportion of individuals with overidentified hypertension and fewer requiring a 2nd measurement; however, this was at the expense of a higher proportion of missed hypertension. Neither is ideal, but overidentified hypertension may be more acceptable than missed hypertension given the limited screening opportunities in low‐ and middle‐income countries. Thus, a lower threshold of 1st BP (eg, 130/80 mmHg) may be preferable.

The adoption of a more complex approach comes with increased risks of overidentified hypertension being made. However, the improvements in missed hypertension rates and efficacy were not evident in all three datasets. Thus, the consideration of the trade‐off between a slightly higher misclassification rate and the simplicity of the approach remains an individualized one for a given setting. In cases where automated devices are available, whether more complex screening approaches could be programmed into those devices remains to be seen.

While the misclassification is certainly of concern, the majority of those who are misclassified are those with BP close to the diagnostic threshold of 140/90 mmHg. We recently reported that higher risk of future cardiovascular diseases was associated with both missed and overidentified hypertension.^13^ The elevated cardiovascular risk in individuals with overidentified hypertension seems to somewhat mitigate the concern of treating these individuals (especially with lifestyle modification). On the other hand, the elevated risk of cardiovascular disease in missed hypertension is concerning since missed hypertension means missed opportunity for treatment. Thus, it is important to establish a system to repeat hypertension screening periodically (eg, annually).

Our study has a few limitations. The BP measurement protocol and the BP device were not uniform across the three datasets. Also, we did not have information on specific device used for each participant in MMM. Moreover, we recognized terminal digit preference in MMM‐Nepal but not in the other two datasets (Figure S5). From another perspective, generally consistent results across these datasets despite different settings appear to indicate the robustness of our findings. Also, the MMM volunteer population likely reflects the type of individual who would approach a health care setting or screening program, and thus our results should be generalized cautiously to entire population. However, these individuals may be the ideal target population to whom these simplified screening approaches might apply. Compared to the NHANES setting, the MMM data reflect a real‐life implementation of a standardized screening protocol and thus may more closely reflect the actual misclassification that might be observed with the implementation of these approaches. Nonetheless, the generalizability of our study should be carefully evaluated since all of NHANES‐USA, MMM‐Nepal, and MMM‐India implemented a standardized BP measurement protocol and provided specific training of BP measurement to their staff.

## 5 CONCLUSIONS

Here we have characterized the implications of several simplified hypertension screening approaches to address the time constraints of large‐scale screening in resource‐limited settings. These findings suggest that alternative approaches utilizing 1st BP supplemented by 2nd BP in some individuals (eg, if 1st BP ≥130/80 mmHg) may ameliorate the time‐intensive screening process with low proportion of misclassification, which can ultimately improve population‐level hypertension detection.

## CONFLICT OF INTEREST

None.

## AUTHOR CONTRIBUTIONS

OT conceptualized the study, drafted the manuscript, performed statistical analysis, revised the manuscript critically for important intellectual content, and gave final approval of the manuscript. MK drafted the manuscript, performed statistical analysis, revised the manuscript critically for important intellectual content, and gave final approval of the manuscript. AM collected the data of MMM‐India, revised the manuscript critically for important intellectual content, and gave final approval of the manuscript. DN collected the data of MMM‐India, revised the manuscript critically for important intellectual content, and gave final approval of the manuscript. YL, EM, TB, CDH, and LA revised the manuscript critically for important intellectual content and gave final approval of the manuscript. KM conceptualized the study, drafted the manuscript, supervised the statistical analysis, revised the manuscript critically for important intellectual content, and gave final approval of the manuscript.

## Supporting information

Supplementary Material

## Data Availability

The NHANES data are publicly available in the following website: https://wwwn.cdc.gov/nchs/nhanes/Search/DataPage.aspx?Component=Examination. The May Measurement Month data may be available upon request to the authors (some restrictions may apply).
