# Supplementary material for: Simplified hypertension screening approaches with low misclassification and high efficiency in the United States, Nepal, and India
Source: J Clin Hypertens (Greenwich). 2021 Sep 3;23(10):1865–71. doi: 10.1111/jch.14299 (PMC8678738; doi:10.1111/jch.14299)

**Title:** Simplified hypertension screening approaches with low misclassification and high efficiency in the United States, Nepal, and India

**Authors:** Olive Tang, PhD,^a,#^, Minghao Kou, MHS,^a,#^, Yifei Lu, MHS,^b^, Edgar R Miller III, MD, PhD.^a^, Tammy Brady, MD,^a^, Cheryl Dennison-Himmelfarb, PhD,^a^, Arun More, MBBS, PGDCC,^c^, Dinesh Neupane, PhD,^a,d^, Lawrence Appel, MD,^a^, Kunihiro Matsushita, MD, PhD,^a,^ *

**Affiliation:** ^a^Johns Hopkins University, Baltimore, MD; ^b^University of North Carolina, Chapel Hill, NC; ^c^Rural Health Progress Trust, Osmanabad, India; ^d^Nepal Development Society, Nepal

***Correspondence author:**

Kunihiro Matsushita, MD, PhD

Department of Epidemiology, Johns Hopkins Bloomberg School of Public Health

Division of Cardiology, Johns Hopkins School of Medicine

Welch Center for Prevention, Epidemiology, and Clinical Research

2024 E. Monument St., Suite 2-600 (Rm 2-602), Baltimore, MD 21287

Tel (443) 287-8766

Fax (410) 367-2384

[kmatsus5@jhmi.edu](mailto:kmatsus5@jhmi.edu)

# These authors contributed equally to this work and should be considered as co-first authors

Supplemental Table I. Adjusted odds ratios (95% confidence intervals) of missed hypertension and overidentified hypertension when using 1^st^ blood pressure only based on multinomial logistic regression, among three age groups: (a) 18-40 years old; (b) 41-59 years old; and (c) 60+ years old.

1. 18-40 years old

|  |  | NHANES-USA |  | MMM-Nepal |  | MMM-India |
| --- | --- | --- | --- | --- | --- | --- |
| Missed hypertension |  |  |  |  |  |  |
| First systolic BP, < 120 mmHg | 8/11003 | ref | 16/4849 |  | 17/1237 | 0 |
| 120 <= sbp1 < 130 mmHg | 37/3256 | 8.23 (3.73, 18.13) | 35/2299 | 2.45 (1.30, 4.61) | 34/792 | 1.74 (0.89, 3.41) |
| 130 <= sbp1 < 140 mmHg | 72-1057 | 42.41 (19.65, 91.52) | 71/1333 | 8.27 (4.49, 15.23) | 43/439 | 3.64 (1.84, 7.19) |
| 140 <= sbp1 < 150 mmHg | 0/219 | - | 0/335 | - | 0/149 | - |
| sbp1 >= 150 mmHg | 0/122 | - | 0/350 | - | 0/112 | - |
| First diastolic BP, < 70 mmHg | 16/8817 | ref | 6/2310 |  | 10/702 | 0 |
| 70 <= dbp1 < 80 mmHg | 21/4816 | 1.15 (0.59, 2.24) | 19/3444 | 1.42 (0.56, 3.62) | 16/1064 | 0.80 (0.35, 1.86) |
| 80 <= dbp1 < 90 mmHg | 80/1690 | 6.91 (3.85, 12.41) | 97/2577 | 5.87 (2.43, 14.18) | 68/702 | 3.92 (1.76, 8.71) |
| 90 <= dbp1 < 100 mmHg | 0/242 | - | 0/586 | - | 0/180 | - |
| dbp1 >= 100 mmHg | 0/92 | - | 0/249 | - | 0/81 | - |
| Overidentified hypertension |  |  |  |  |  |  |
| First systolic BP, < 120 mmHg | 26/11003 |  | 41/4849 |  | 11/1237 | 0 |
| 120 <= sbp1 < 130 mmHg | 55/3256 | 3.13 (1.86, 5.25) | 137/2299 | 4.31 (2.95, 6.32) | 13/792 | 1.94 (0.81, 4.62) |
| 130 <= sbp1 < 140 mmHg | 52/1057 | 4.15 (2.34, 7.34) | 148/1333 | 5.07 (3.40, 7.56) | 52/439 | 8.50 (3.88, 18.61) |
| 140 <= sbp1 < 150 mmHg | 94/219 | 51.54 (29.66, 89.56) | 355/335 | 59.65 (40.25, 88.41) | 70/149 | 28.51 (12.80, 63.49) |
| sbp1 >= 150 mmHg | 1/122 | 0.27 (0.03, 2.08) | 67/350 | 6.52 (4.06, 10.48) | 20/112 | 12.15 (4.84, 30.51) |
| First diastolic BP, < 70 mmHg | 26/8817 |  | 29/2310 |  | 11/702 | 0 |
| 70 <= dbp1 < 80 mmHg | 31/4816 | 1.68 (0.98, 2.89) | 94/3444 | 1.36 (0.87, 2.13) | 22/1064 | 0.82 (0.37, 1.80) |
| 80 <= dbp1 < 90 mmHg | 29/1690 | 2.64 (1.46, 4.75) | 180/2577 | 2.01 (1.29, 3.12) | 34/702 | 0.94 (0.42, 2.10) |
| 90 <= dbp1 < 100 mmHg | 141/242 | 81.63 (47.76, 139.52) | 400/586 | 19.85 (12.64, 31.17) | 91/180 | 5.03 (2.25, 11.23) |
| dbp1 >= 100 mmHg | Jan-92 | 0.50 (0.06, 3.91) | 45/249 | 3.22 (1.83, 5.68) | 8/81 | 0.88 (0.30, 2.62) |

1. 41-59 years old

|  |  | NHANES-USA |  | MMM-Nepal |  | MMM-India |
| --- | --- | --- | --- | --- | --- | --- |
| Missed hypertension |  |  |  |  |  |  |
| First systolic BP, < 120 mmHg | 6/4071 | ref | 10/1169 | ref | 10/484 | ref |
| 120 <= sbp1 < 130 mmHg | 25/2122 | 5.33 (2.16, 13.19) | 20/731 | 1.87 (0.83, 4.21) | 23/501 | 0.98 (0.43, 2.27) |
| 130 <= sbp1 < 140 mmHg | 83/1212 | 30.19 (12.87, 70.78) | 37/539 | 5.62 (2.62, 12.05) | 34/340 | 2.25 (0.99, 5.11) |
| 140 <= sbp1 < 150 mmHg | 0/392 | - | 0/320 | - | 0/176 | - |
| sbp1 >= 150 mmHg | 0/488 | - | 0/374 | - | 0/277 | - |
| First diastolic BP, < 70 mmHg | 7/2529 | ref | 2/386 | ref | 2/192 | ref |
| 70 <= dbp1 < 80 mmHg | 33/3309 | 2.06 (0.90, 4.72) | 9/957 | 1.17 (0.24, 5.64) | 10/568 | 1.62 (0.35, 7.59) |
| 80 <= dbp1 < 90 mmHg | 74/1884 | 5.34 (2.40, 11.89) | 56/1016 | 5.47 (1.23, 24.30) | 55/616 | 7.81 (1.72, 35.39) |
| 90 <= dbp1 < 100 mmHg | 0/426 | - | 0/480 | - | 0/252 | - |
| dbp1 >= 100 mmHg | 0/137 | - | 0/294 | - | 0/150 | - |
| Overidentified hypertension |  |  |  |  |  |  |
| First systolic BP, < 120 mmHg | 16/4071 | ref | 18/1169 | ref | 5/484 | ref |
| 120 <= sbp1 < 130 mmHg | 67/2122 | 6.26 (3.57, 10.98) | 38/731 | 1.95 (1.06, 3.58) | 12/501 | 1.89 (0.62, 5.74) |
| 130 <= sbp1 < 140 mmHg | 69/1212 | 7.75 (4.35, 13.82) | 71/539 | 3.40 (1.88, 6.16) | 31/340 | 5.32 (1.86, 15.24) |
| 140 <= sbp1 < 150 mmHg | 258/392 | 104.47 (60.58, 180.13) | 156/320 | 13.33 (7.46, 23.82) | 76/176 | 21.46 (7.54, 61.08) |
| sbp1 >= 150 mmHg | 15/488 | 2.66 (1.24, 5.72) | 23/374 | 1.95 (0.96, 3.99) | 21/277 | 4.80 (1.56, 14.79) |
| First diastolic BP, < 70 mmHg | 38/2529 | ref | 5/386 | ref | 3/192 | ref |
| 70 <= dbp1 < 80 mmHg | 88/3309 | 1.04 (0.69, 1.58) | 22/957 | 1.23 (0.45, 3.38) | 15/568 | 1.13 (0.31, 4.15) |
| 80 <= dbp1 < 90 mmHg | 124/1884 | 1.32 (0.88, 2.00) | 89/1016 | 2.70 (1.02, 7.14) | 39/616 | 1.43 (0.39, 5.16) |
| 90 <= dbp1 < 100 mmHg | 175/426 | 9.20 (5.93, 14.27) | 181/480 | 8.03 (3.01, 21.40) | 86/252 | 4.54 (1.25, 16.46) |
| dbp1 >= 100 mmHg | 0/137 | - | 9/294 | 0.62 (0.19, 2.01) | 2/150 | 0.22 (0.03, 1.47) |

1. 60+ years old

|  |  | NHANES-USA |  | MMM-Nepal |  | MMM-India |
| --- | --- | --- | --- | --- | --- | --- |
| Missed hypertension |  |  |  |  |  |  |
| First systolic BP, < 120 mmHg | 1/1358 | ref | 2/316 | ref | 7/312 | ref |
| 120 <= sbp1 < 130 mmHg | 14/1178 | 12.96 (1.70, 99.06) | 5/227 | 2.64 (0.49, 14.29) | 17/278 | 2.90 (1.14, 7.42) |
| 130 <= sbp1 < 140 mmHg | 92/1047 | 89.56 (12.39, 647.53) | 19/204 | 13.69 (2.97, 63.11) | 37/287 | 6.54 (2.67, 16.02) |
| 140 <= sbp1 < 150 mmHg | 0/450 | - | 0/113 | - | 0/205 | - |
| sbp1 >= 150 mmHg | 0/1148 | - | 0/266 | - | 0/494 | - |
| First diastolic BP, < 70 mmHg | 33/2438 | ref | 1/167 | ref | 12/234 | ref |
| 70 <= dbp1 < 80 mmHg | 38/1711 | 1.51 (0.92, 2.48) | 8/319 | 2.50 (0.30, 21.10) | 20/475 | 0.64 (0.29, 1.42) |
| 80 <= dbp1 < 90 mmHg | 36/757 | 3.50 (2.06, 5.92) | 17/340 | 4.02 (0.49, 32.83) | 29/475 | 0.98 (0.44, 2.15) |
| 90 <= dbp1 < 100 mmHg | 0/224 | - | 0/202 | - | 0/264 | - |
| dbp1 >= 100 mmHg | 0/51 | - | 0/98 | - | 0/128 | - |
| Overidentified hypertension |  |  |  |  |  |  |
| First systolic BP, < 120 mmHg | 1/1358 | ref | 4/316 | ref | 1/312 | ref |
| 120 <= sbp1 < 130 mmHg | 12/1178 | 14.42 (1.87, 111.10) | 11/227 | 3.49 (1.05, 11.62) | 9/278 | 11.00 (1.36, 89.01) |
| 130 <= sbp1 < 140 mmHg | 14/1047 | 19.11 (2.51, 145.75) | 8/204 | 2.43 (0.68, 8.71) | 16/287 | 20.14 (2.58, 157.06) |
| 140 <= sbp1 < 150 mmHg | 417/450 | 1359.87 (189.96, 9734.86) | 65/113 | 39.77 (12.92, 122.36) | 103/205 | 206.36 (27.48, 1549.91) |
| sbp1 >= 150 mmHg | 56/1148 | 71.91 (9.88, 523.33) | 13/266 | 3.31 (0.95, 11.51) | 26/494 | 25.10 (3.24, 194.43) |
| First diastolic BP, < 70 mmHg | 180/2438 |  | 5/167 |  | 11/234 |  |
| 70 <= dbp1 < 80 mmHg | 181/1711 | 0.86 (0.66, 1.14) | 14/319 | 0.84 (0.27, 2.61) | 40/475 | 0.67 (0.31, 1.48) |
| 80 <= dbp1 < 90 mmHg | 93/757 | 0.76 (0.54, 1.05) | 37/340 | 1.22 (0.42, 3.60) | 62/475 | 0.78 (0.36, 1.68) |
| 90 <= dbp1 < 100 mmHg | 46/224 | 1.23 (0.78, 1.94) | 43/202 | 2.14 (0.71, 6.44) | 37/264 | 0.55 (0.24, 1.25) |
| dbp1 >= 100 mmHg | 0/51 | - | Feb-98 | 0.14 (0.02, 0.84) | 5/128 | 0.23 (0.07, 0.76) |

Supplemental Figure I. Flow Chart of Study Population Selection in NHANES-USA, MMM-Nepal, and MMM-India


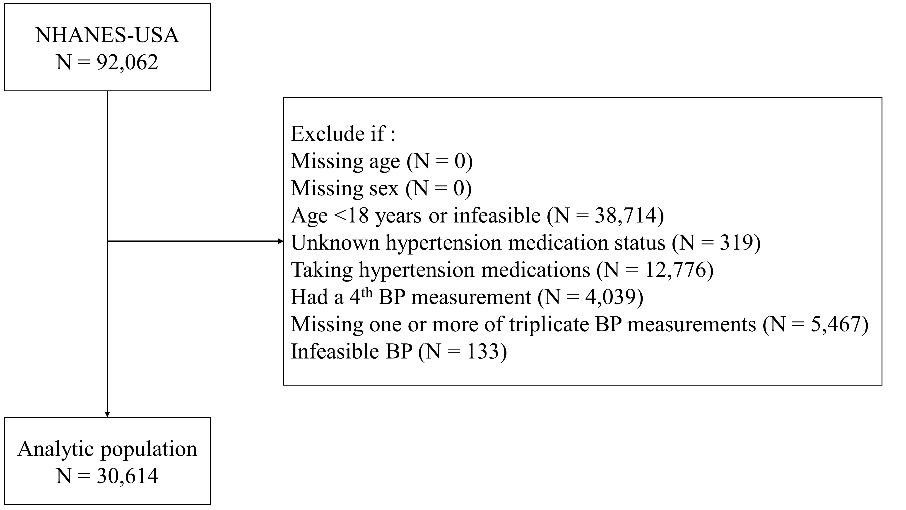

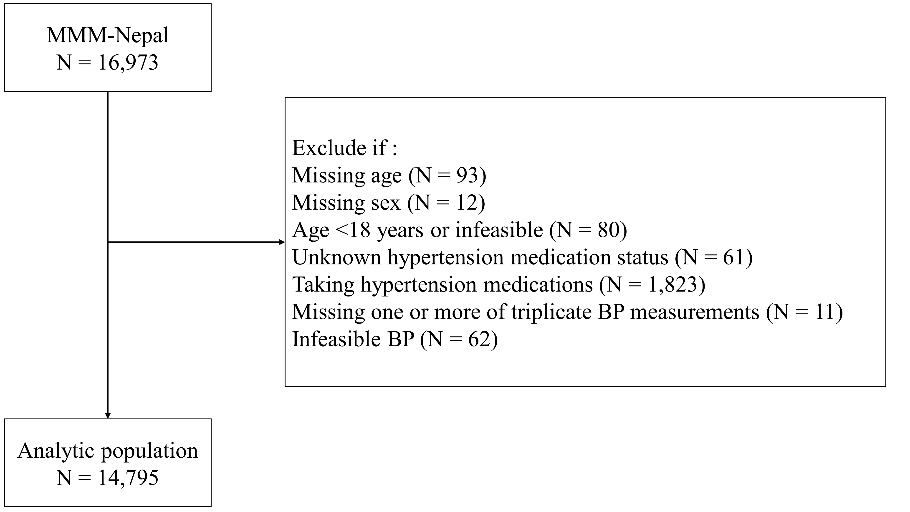

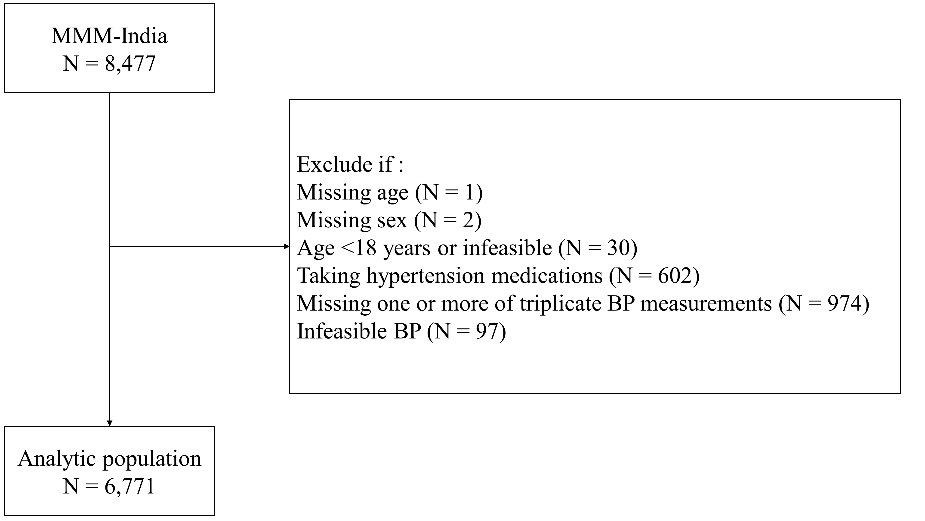


Supplemental Figure II**.** Hypertension prevalence according to first systolic blood pressure and diastolic blood pressure measurement. Hypertension was defined as the average of 2^nd^ and 3^rd^ SBP≥140 and/or DBP≥90 mmHg. Distribution of first SBP, and hypertension prevalence in NHANES-USA (A), MMM-Nepal (B) and MMM-India (C). Distribution of first DBP, and hypertension prevalence in NHANES-USA (D), MMM-Nepal (E) and MMM-India (F). DBP indicates diastolic blood pressure; MMM, May Measurement Month; NHANES, National Health and Nutrition Examination Survey; SBP, systolic blood pressure.

A
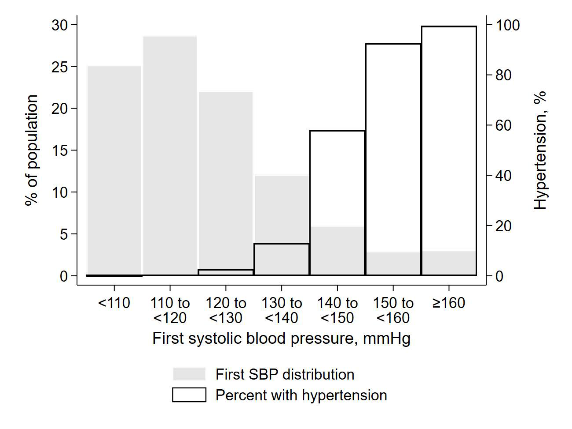
 B
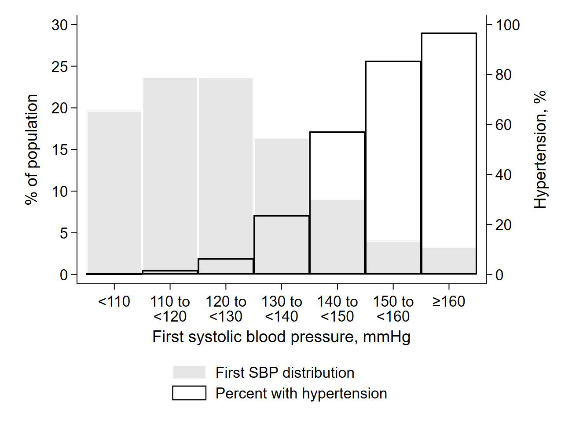


C
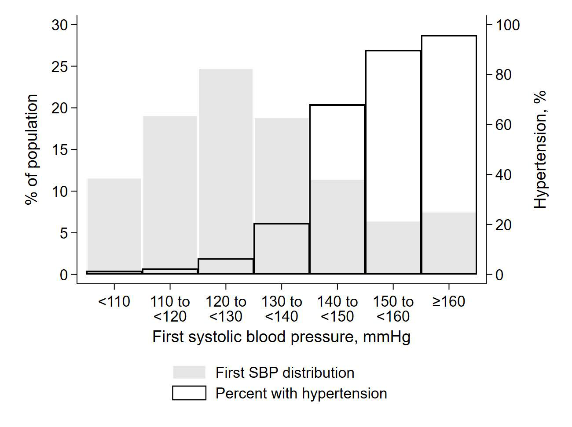
 D
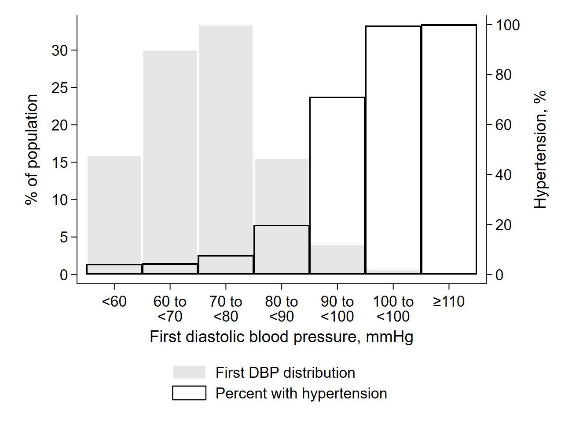


E
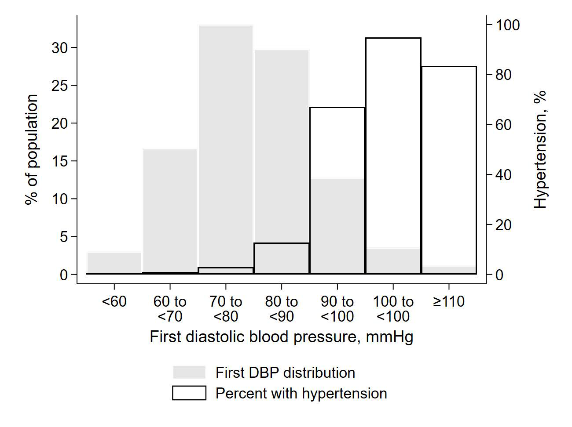
 F
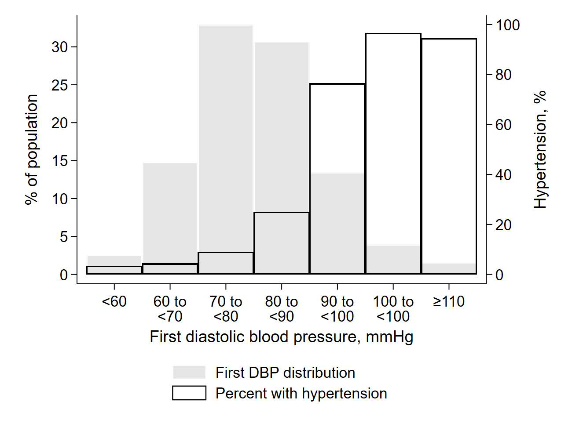
Supplemental Figure III. Missed hypertension and overidentified hypertension distribution in the standard definition. Distribution of missed and overidentified hypertension by the first systolic blood pressure in NHANES-USA (A), MMM-Nepal (B) and MMM-India (C). Distribution of missed and overidentified hypertension by the first diastolic blood pressure measurement in NHANES-USA (D), MMM-Nepal (E) and MMM-India (F). MMM indicates May Measurement Month; NHANES, National Health and Nutrition Examination Survey.

A
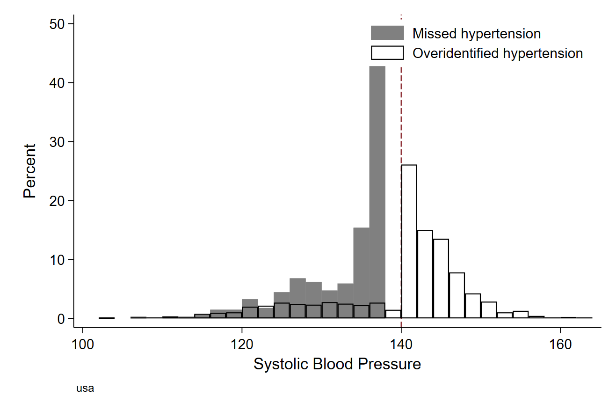
 B
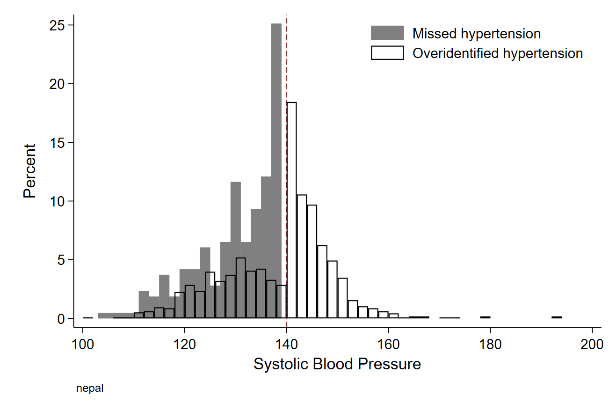


C
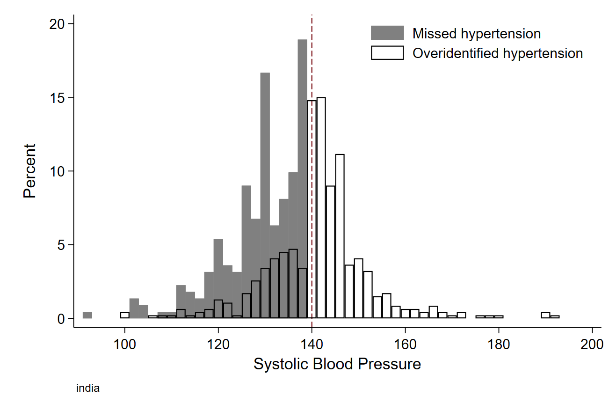
 D
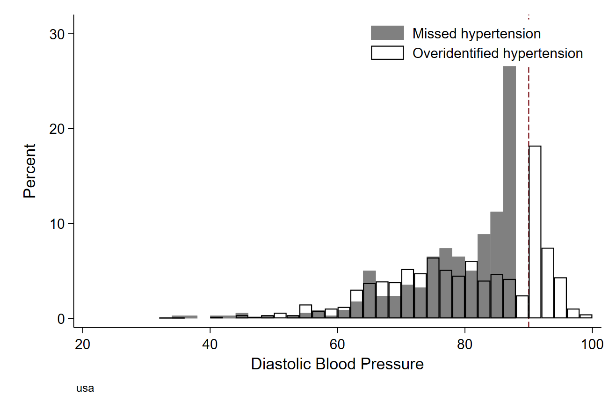


E
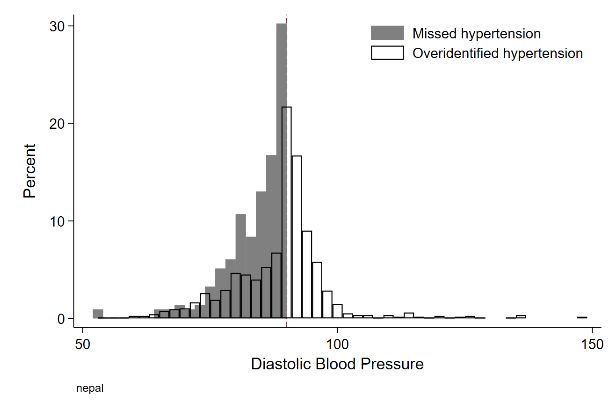
 F
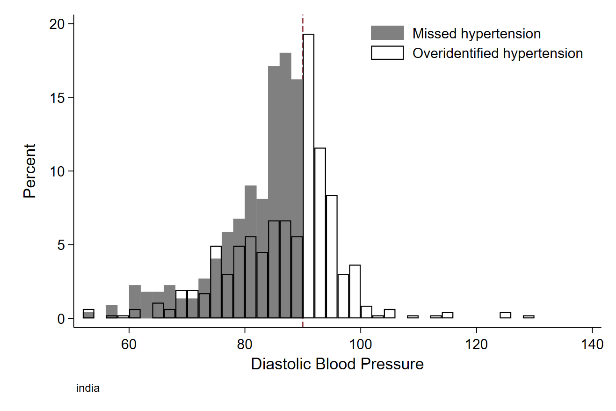


Supplemental Figure IV. Misclassification and efficiency by the simplified approaches vs. standard approach, stratified by age (≤50, and > 50 years old). Color bars indicate degree of misclassification, the proportion of individuals requiring 2^nd^ BP measurement (out of 100%), or average number of measurements required per person (3 is the standard approach). BP indicates blood pressure; MMM, May Measurement Month; NHANES, National Health and Nutrition Examination Survey.


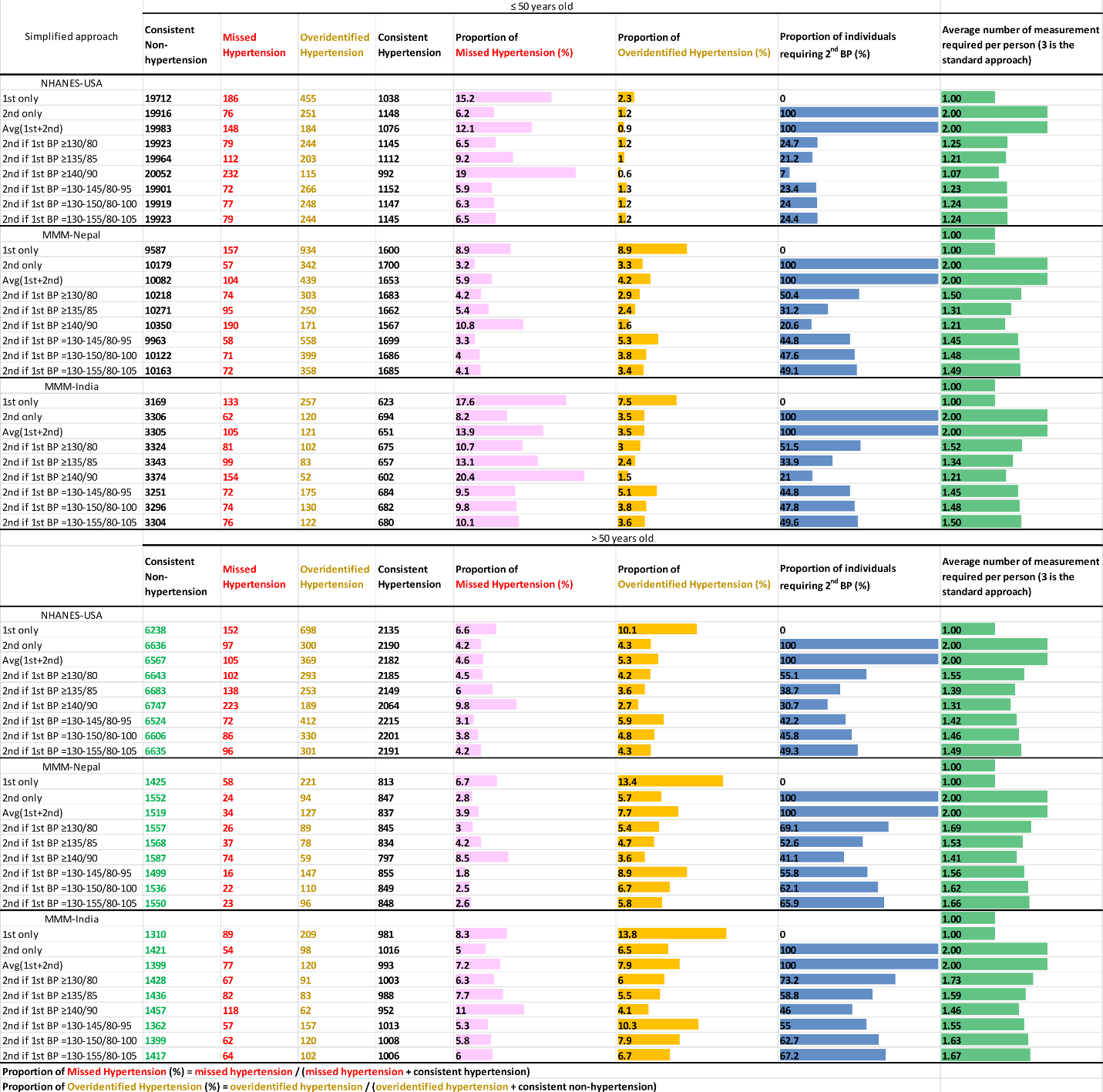


Supplemental Figure V. Distribution of terminal digits across NHANES-USA (a), MMM-Nepal (b), and MMM-India (c) for 1^st^ BP (left column), 2^nd^ BP (middle column), and 3^rd^ BP (right column).

1. NHANES-USA


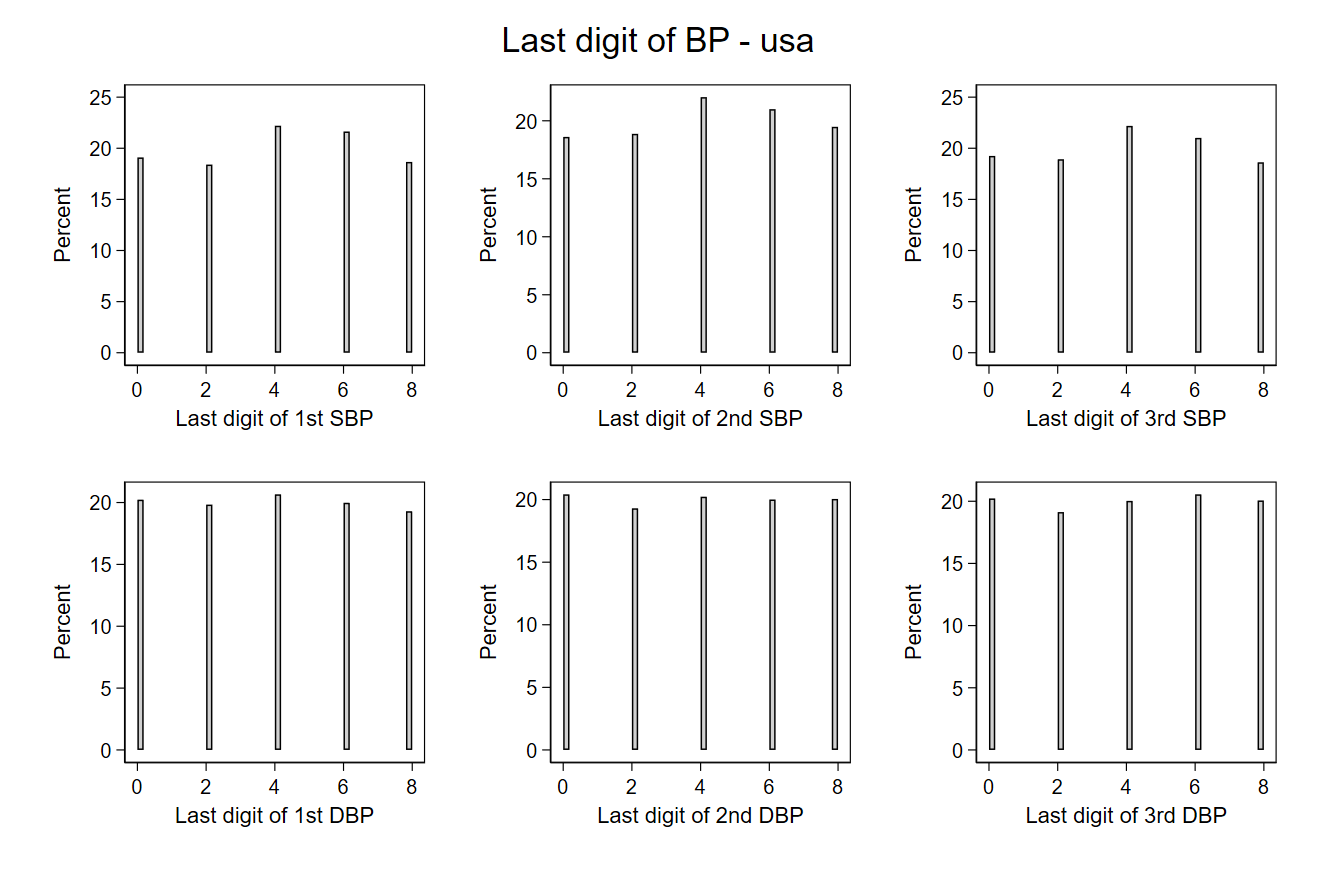


1. MMM-Nepal


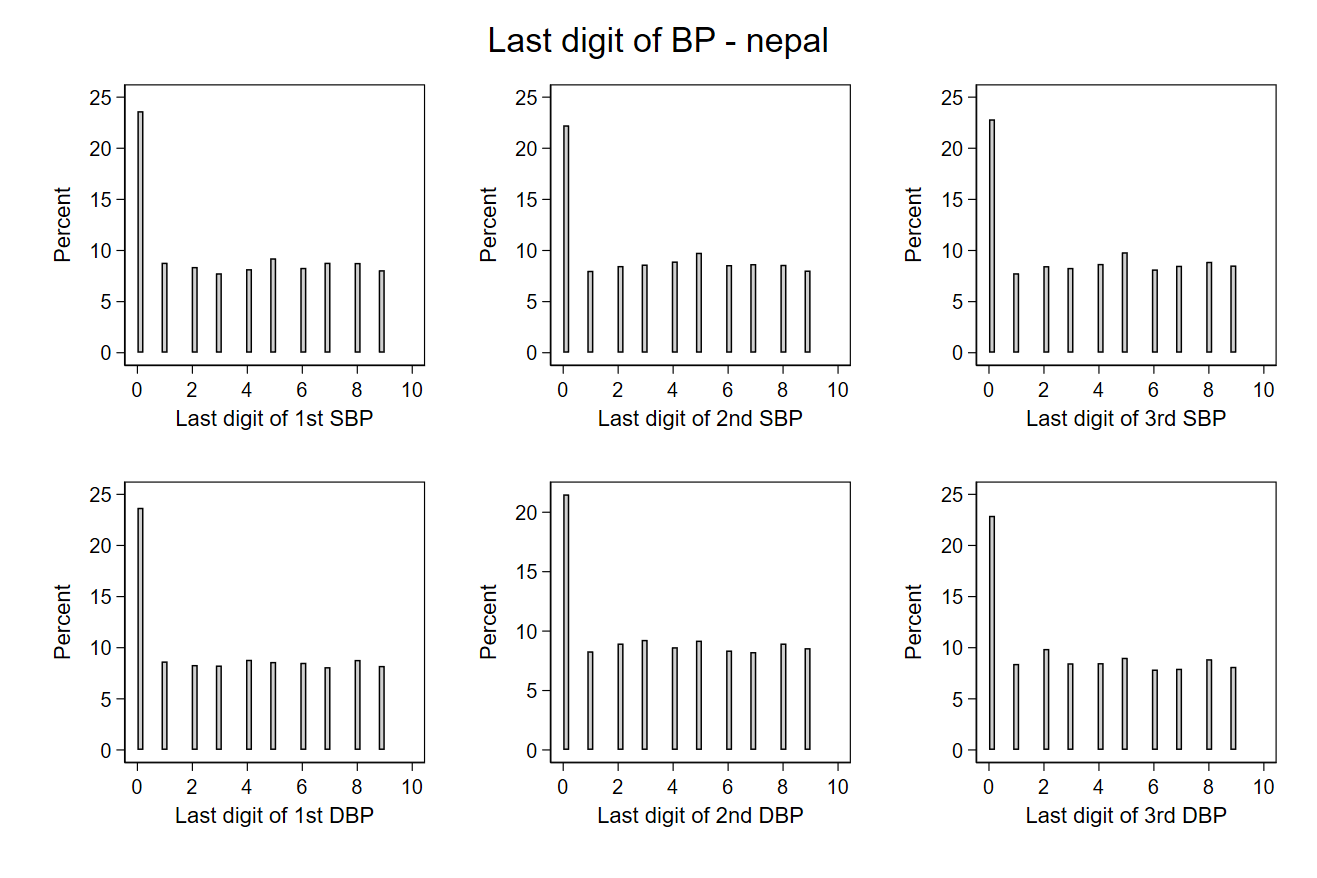


1. MMM-India


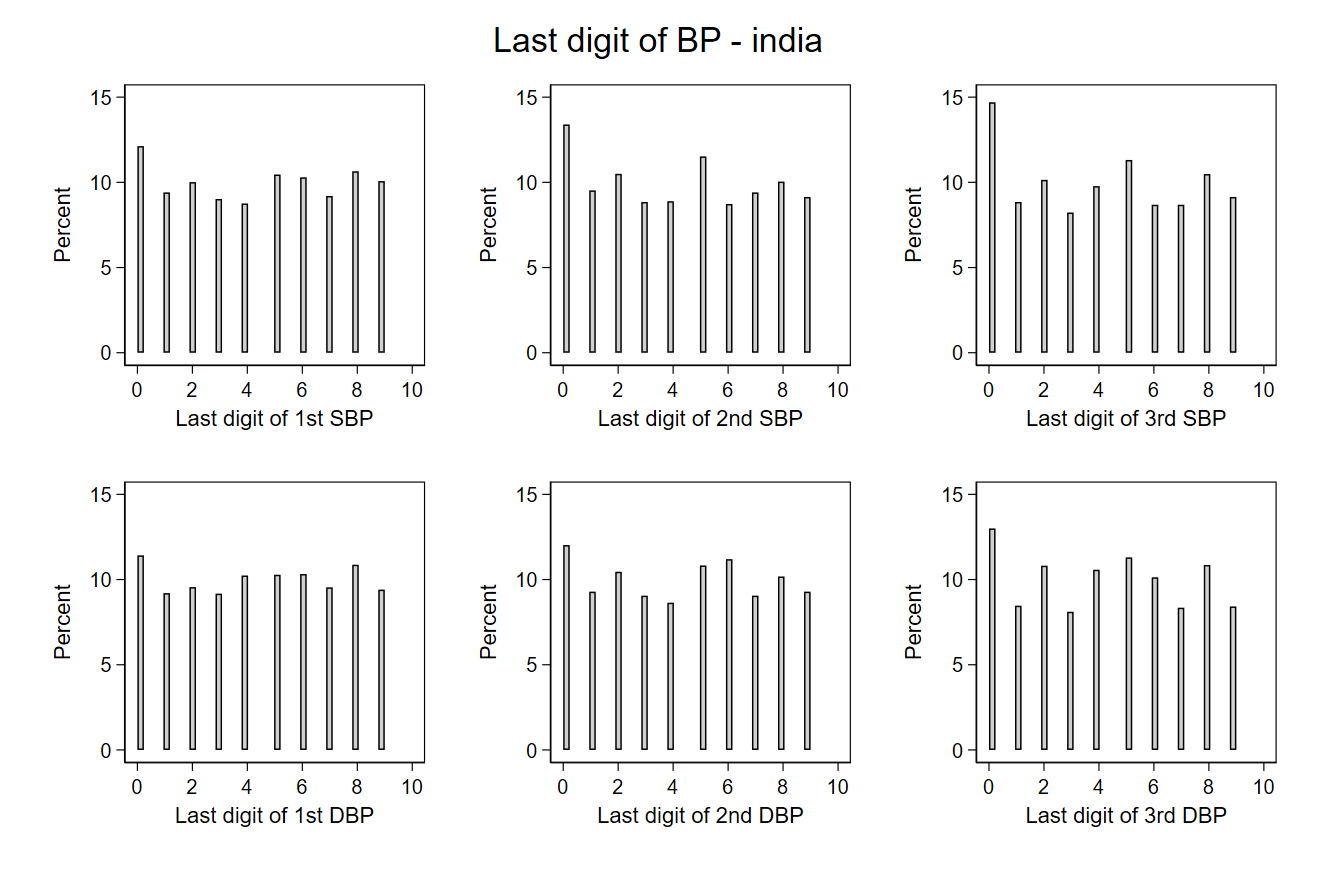

Supplement: Supplementary file 1 — Supplementary Material [file JCH-23-1865-s001.docx]
